# Renal effects of growth hormone in health and in kidney disease

**DOI:** 10.1007/s00467-021-05097-6

**Published:** 2021-06-18

**Authors:** Dieter Haffner, Andrea Grund, Maren Leifheit-Nestler

**Affiliations:** 1 Department of Pediatric Kidney, Liver and Metabolic Diseases, Pediatric Research Center, Hannover Medical School, Carl-Neuberg-Str. 1, 30625 Hannover, Germany; 2 Pediatric Research Center, Hannover Medical School, Carl-Neuberg-Str. 1, 30625 Hannover, Germany

**Keywords:** Growth hormone, IGF-1, Klotho, Hypertrophy, Children, Chronic kidney disease, Nephrotic syndrome, Diabetic nephropathy

## Abstract

Growth hormone (GH) and its mediator insulin-like growth factor-1 (IGF-1) have manifold effects on the kidneys. GH and IGF receptors are abundantly expressed in the kidney, including the glomerular and tubular cells. GH can act either directly on the kidneys or via circulating or paracrine-synthesized IGF-1. The GH/IGF-1 system regulates glomerular hemodynamics, renal gluconeogenesis, tubular sodium and water, phosphate, and calcium handling, as well as renal synthesis of 1,25 (OH)_2_ vitamin D_3_ and the antiaging hormone Klotho. The latter also acts as a coreceptor of the phosphaturic hormone fibroblast-growth factor 23 in the proximal tubule. Recombinant human GH (rhGH) is widely used in the treatment of short stature in children, including those with chronic kidney disease (CKD). Animal studies and observations in acromegalic patients demonstrate that GH-excess can have deleterious effects on kidney health, including glomerular hyperfiltration, renal hypertrophy, and glomerulosclerosis. In addition, elevated GH in patients with poorly controlled type 1 diabetes mellitus was thought to induce podocyte injury and thereby contribute to the development of diabetic nephropathy. This manuscript gives an overview of the physiological actions of GH/IGF-1 on the kidneys and the multiple alterations of the GH/IGF-1 system and its consequences in patients with acromegaly, CKD, nephrotic syndrome, and type 1 diabetes mellitus. Finally, the impact of short- and long-term treatment with rhGH/rhIGF-1 on kidney function in patients with kidney diseases will be discussed.

## Introduction

**Fig. 1:**
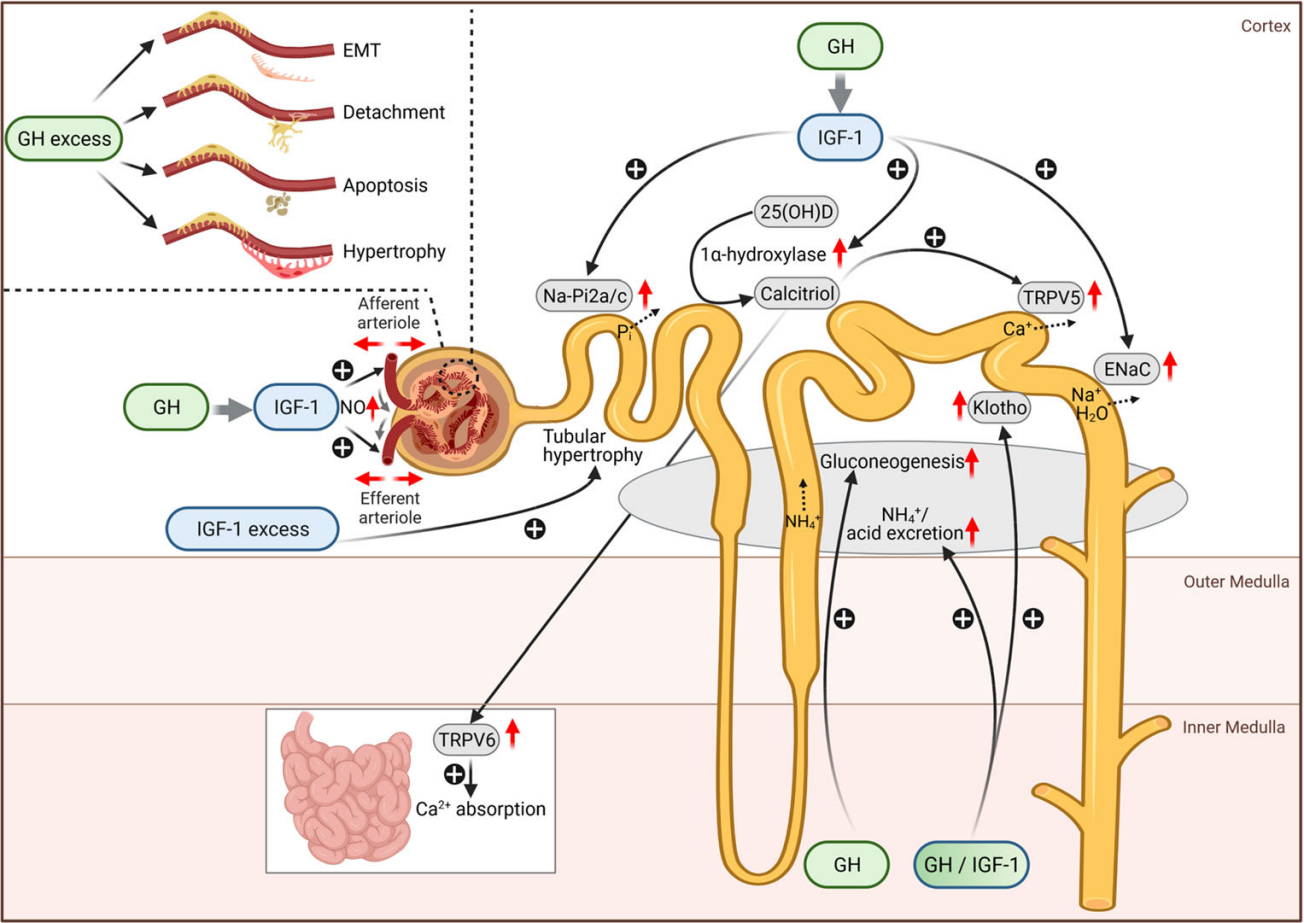
Physiological and pathophysiological actions of growth hormone and its mediator insulin-like growth factor-1 (IGF-1) on the kidneys: GH and IGF-1 receptors (GHR/IGF-1R) are widely expressed in the glomerular and tubular cells. GH can act on the kidneys, either directly via activation of the GHR/Janus kinase-2 (JAK2)-signal transducer and activator of transcription 5 (STAT5) and ERK1/2 pathways, or indirectly via circulating or paracrine-synthesized IGF-1 by activating IGF-1R. Most of the glomerular and tubular effects of GH are mediated by IGF-1 and include the following: (1) dilation of the afferent and efferent arterioles via increased synthesis of the endogenous vasodilator nitric oxide (NO), resulting in enhanced glomerular filtration rate and renal plasma flow; (2) stimulation of phosphate (Pi) reabsorption in the proximal tubules via up-regulation of the sodium-phosphate transporters (Na-Pi2a/2c; (3) stimulation of sodium (Na^+^) and water (H_2_O) reabsorption in the distal nephron via up-regulation of the epithelial sodium channel (ENaC); and (4) stimulation of the 1α-hydroxylase and thereby, calcitriol synthesis in the proximal tubule, with subsequent increases in calcium (Ca^+^) absorption via up-regulation of the epithelial calcium channels TRPV6 and TRPV5 in the intestine and distal renal tubule, respectively. Some GH effects may also be mediated by IGF-1, including stimulation of (1) net acid secretion via increased ammonia (NH_4_^+^) production in the proximal tubule and a Na^+^-dependent mechanism in the distal tubule and (2) renal Klotho synthesis in the distal renal tubule. GH was shown to directly enhance renal gluconeogenesis in proximal tubular cells. In states of GH excess, GH can directly induce glomerulosclerosis and podocyte injury, characterized by podocyte hypertrophy, apoptosis, dedifferentiation of podocytes (epithelial–mesenchymal transition, EMT), and/or cross-linking of the basement membrane resulting in increased podocyte permeability to albumin and detachment of podocytes from the glomerular basement membrane. By contrast, IGF-1 excess results in tubular hypertrophy only. The kidneys of patients with chronic kidney disease (CKD) are protected from the potentially negative effects of long-term GH-treatment on the glomerulus, likely due to the CKD-associated GH insensitivity of the kidneys and/or the much lower GH exposure in GH-treated patients, compared to those with the abovementioned conditions.

Growth hormone (GH) is widely used for the treatment of short stature in children, including those suffering from chronic kidney disease (CKD). The GH / insulin-like growth factor-1 (IGF-1) system has profound effects on the kidneys, including glomerular and tubular function, as well as the synthesis of 1,25 (OH)_2_ vitamin D_3_ and the antiaging hormone Klotho (Fig. 1). Animal studies and observations in acromegalic patients have demonstrated that GH-excess can impact on kidney health, including glomerular hyperfiltration and hypertrophy and glomerulosclerosis. In addition, elevated GH in patients with poorly controlled type 1 diabetes mellitus was shown to induce podocyte injury and thereby may contribute to diabetic nephropathy. This manuscript gives an overview of the physiological actions of GH and IGF-1 on the kidneys and summarizes the current knowledge of their impact on kidney health in subjects with normal and impaired kidney function, including acute kidney injury (AKI), CKD and nephrotic syndrome. We will update and expand on previous, excellent reviews on this topic published over the last three decades [1–5].

## Physiology of GH

### GH and IGFs

Growth hormone is a 22-kDa protein, which is secreted from the anterior pituitary gland in a pulsatile, predominantly nocturnal fashion, with highest secretion rates during puberty [6]. In the circulation, GH is bound to the high affinity GH binding protein (GHBP), which is generated by proteolytic cleavage of the cell membrane-bound GH receptor (GHR). GHBP modulates the actions of GH, for instance, by increasing its half-life or acting as a negative regulator of receptor signaling via dimerization with the GHR. GH is stimulated by the hypothalamic GH-releasing hormone (GHRH) and inhibited by somatostatin. It plays a major role in postnatal growth and is involved in many other biological functions, including metabolism and homeostasis. GH acts on target tissues mainly via stimulation of the somatomedin insulin-like growth factor-1 (IGF-1, 7.6 kDa), and to a lesser extent, directly in an IGF-1 independent manner. Within the circulation, more than 99% of IGFs are bound to specific high-affinity IGF-binding proteins (IGFBPs), six of which have been identified, regulating their half-life and bioavailability [7]. IGFBP-3 (60 kDa) is the predominant IGFBP in the circulation after the neonatal period. It associates with an acid-labile subunit (ALS, 85 kDa) and IGF-1 or -2, forming a high-molecular weight ternary complex of approx. 150 kDa which functions as a reservoir of IGFs, thereby keeping the serum concentrations of free IGFs constant. The second and third most abundant IGFBPs are IGFBP-2 (32 kDa) and IGBP-1 (28 kDa), which can also bind IGF-1 and IGF-2, and are thought to modulate IGF actions by competing with the IGF receptors.

GH and IGF-1 act synergistically with respect to growth and the kidneys and antagonistically on glucose metabolism, i.e., GH stimulates glucose synthesis and concentrations, whereas IGF-1 decreases serum glucose levels. After binding to the GHR, the intracellular Janus kinase-2 (JAK2)-signal transducer and activator of transcription 5 (STAT5) and ERK1/2 pathways are activated [8]. In addition, proteins of the suppressor cytokine signaling (SOCS) family are synthesized, acting as a negative feedback mechanism. IGF-1 exerts its actions on a cellular level via the IGF-1 receptor (IGF-1R) which results in activation of several intracellular pathways, including the ERK1/2 and phosphatidylinositol 3-kinase/AKT pathways.

### GH and IGF signaling in the kidney

#### GH receptor

The GHR is expressed in most tissues, including the kidney. In 1989 GHR mRNA in the rat was already shown to be mainly expressed in the straight proximal tubules from embryonic day 20 onwards, increasing until postnatal day 40 and with constant levels thereafter [9]. A much more widespread expression of GHR was revealed by immunostaining in human fetal kidneys, including all nephron segments. By contrast, only a very weak signal was detected in the glomeruli of fetal but not in adult kidneys [10]. The latter suggests that GH may be involved in glomerular morphogenesis. Recent investigations in rodents and humans confirmed GHR expression in all tubular segments and this extended to the predominant glomerular cell types, i.e., mesangial cells and podocytes, when using quantitative real-time RT-PCR techniques [11–14]. The concept of direct GH action on glomerular cells is supported by studies in transgenic mice overexpressing human or bovine GH showing progressive glomerulosclerosis, whereas IGF-1 overexpressing mice lack glomerular changes [14]. Several studies confirmed the integrity of GH-signaling via GHR/JAK2/STAT5 pathway in the kidney by using renal cell lines [11, 12].

#### Insulin-like growth factor-1 and 2

Renal IGF-1 not only derives from the circulation (mainly synthesized in the liver) but is also locally produced in the kidney [15]. It exerts its paracrine and autocrine effects under the regulation of circulating GH [16]. While IGF-1 was shown to be expressed in all renal cells during fetal life, a drastic decrease was noted after birth. After birth, IGF-1 synthesis is restricted to the medullary, thick ascending limb of Henle’s loop, collecting ducts, and peritubular capillaries of the outer medulla and inner cortex [10, 17–19]. The distribution of IGF-1 and its availability in kidney tissues is under the regulation of six high-affinity IGF binding proteins (IGFBPs) [20]. IGF-2 is also highly expressed in the fetal kidney and plays an important role during kidney development, whereas its role after birth needs to be clarified [21].

#### IGF-1 receptor

IGF-1 receptor (IGF-1R) was shown to be expressed in both rodents and humans throughout the nephron, including the glomeruli [10, 19, 22–24]. Whereas initial studies suggest that its expression in the proximal tubules is very low, recent studies using microdissected, freshly-isolated, murine, nephronic segments demonstrated the highest IGF-1R mRNA expression in proximal tubules along the nephron [11]. The functional integrity of IGF-1-induced IGF-1R signaling in the kidney was demonstrated by using renal cell lines [11, 25, 26].

### GH and kidney development

GH knockout mice (GH^-/-^) show reduced kidney weight compared to wild-type mice, even after correction for reduced body weight [27]. Unfortunately, studies on renal histology and glomerular function in GH^-/-^ mice are lacking. In contrast to GH^-/-^ mice, IGF-1^-/-^ mice show a proportional reduction in kidney weight when compared to wild-type mice. However, histological studies revealed slightly smaller glomeruli and a 20% reduction in the number of glomeruli in IGF-1^-/-^ mice compared to controls [3]. The contribution of circulating versus locally produced IGF-1 in kidney development was investigated using a hepatic specific JAK2 knockout in mice [28]. As expected, hepatic specific JAK2^-/-^ mice showed no hepatic IGF-1 synthesis and markedly reduced IGF-1 serum concentrations compared to wild-type mice. In addition, kidney weight was markedly reduced in GH-treated and untreated JAK2 ^-/-^ mice compared to controls, indicating the importance of circulating IGF-1 in kidney growth, whereas locally produced IGF-1 seems not to have a significant impact on kidney growth.

### GH and renal hemodynamics

Corvilain et al. demonstrated in their seminal study that exogenous GH, i.e., GH-containing pituitary extracts, increases glomerular filtration rate (GFR) in healthy subjects [29]. This could be confirmed by using daily subcutaneous injections of recombinant human GH (rhGH), resulting in increases ranging from 11–18% compared to baseline after three and up to 7 days of treatment [30–33]. Equally, (rh)GH was shown to increase renal plasma flow (RPF) in healthy subjects by approx. 25% after 3 and up to 7 days of treatment [30–32, 34]. However, GH-infusion in healthy subjects had no effects on GFR or RPF over a 2-h observation period [35]. Serial GFR and RPF measurements in a GH-deficient adult revealed a delayed rhGH-induced rise in GFR and RPF, starting 24 h after injection, in parallel with the concomitant rise in IGF-1 serum concentrations [31]. Similar findings were later seen in healthy subjects [30]. Taken together, these studies suggest that the GH-induced rise in GFR and RPF is mediated by IGF-1, rather than due to the direct effects of GH on the glomerulus [2]. Subsequent studies in healthy subjects and rodents revealed that application of IGF-1 leads to an acute rise (< 20 min) in GFR and RPF of approximately 20–30%, compared to baseline values [36–38]. These effects were maintained during a 7-day IGF-1 treatment in rats [39]. Using micropuncture studies it was shown that these effects are mainly due to dilation of both the efferent and afferent arterioles and resulted in reduction of renal vascular resistance, as illustrated in Fig. 1 [40, 41]. The effects of GH on GFR and RPF in humans could be blunted by coadministration of cyclooxygenase inhibitors, suggesting that they depend on the availability of vasodilating prostaglandins [42]. Also, the vasodilatory effects of IGF-1 on renal juxtamedullary microvasculature could be completely blocked by pretreatment with the cyclooxygenase inhibitor indomethacin [41]. Moreover, there is evidence that the vasodilatory effects of IGF-1 are mediated by nitric oxide (NO) and cyclic GMP (cGMP). Studies in cultured, vascular, endothelial cells showed that IGF-1 induces NO release [43]. Administration of IGF-1 in rats increases urinary excretion of cGMP which mediates the intracellular effects of NO on smooth muscle cells [2]. More importantly, the acute IGF-1-induced rise in GFR and RPF in rodents is blunted by coadministration of an NO synthase inhibitor [42]. Taken together, the effects of IGF-1 on renal vasodilation depend on the synthesis of endogenous vasodilators including NO and prostaglandins.

Interestingly, GH also has hemodynamic effects in nonrenal vascular beds. A 7-day treatment with rhGH significantly increased microvascular blood flow, as assessed by nailfold capillaroscopy and leg-strain gauge plethysmography in healthy subjects [44]. This was associated with a reduction of total vascular resistance, and increased heart rate and cardiac index. These effects are also most likely mediated by IGF-1.

### GH and glomerular cells

Reddy et al. identified the glomerular podocyte as a target for GH action by demonstrating that cultured murine podocytes and kidney glomeruli express GHR and treatment with rhGH, of both murine and human podocytes, results in activation of the GHR/JAK2/STAT5 and ERK1/2 pathways in these cells [12]. In addition, treatment with rhGH stimulated the focal adhesion kinase, increased reactive oxygen species and reorganization of the podocyte actin cytoskeleton [12]. The same group also demonstrated that zinc finger E-box-binding, homebox 2 (ZEB2), is upregulated in immortalized human podocytes by rhGH-treatment in a dose- and time-dependent manner [45]. The GH-induced increase in ZEB2 expression caused a downregulation of E- and P-cadherins. In addition, the GH-induced increase in podocyte permeability to albumin in a paracellular albumin influx assay could be completely blocked by a ZEB2 knockdown, suggesting that exposure to high levels of GH may induce glomerular albuminuria, due to increased podocyte permeability (*vide infra*) [45]. However, both GH and IGF-1 were also shown to have positive effects on the glomerulus. Epithelial-to-mesenchymal-transition, which is an important component in the development of glomerulosclerosis, could be completely reversed by administration of rhGH in a model of hyperhomocystinemia-induced podocyte dysfunction [46]. Treatment with IGF-1 resulted in enhanced cell migration and differentiation and, thereby, ameliorated podocyte apoptosis induced by cytokine or high glucose concentration [47]. Therefore, the physiological role of GH and IGF-1 on glomerular function is still unclear.

### GH and tubular function

#### Phosphate

Phosphate hemostasis is highly regulated by the kidneys. Under normal conditions, approx. 80% of filtered phosphate is reabsorbed in the renal proximal tubules by sodium-phosphate transporters (Na-Pi2a and 2c) under the regulation of hormones, including parathyroid hormone (PTH), fibroblast-growth factor 23 (FGF23), and IGF-1 [48]. The physiological increase in renal phosphate handling occurring during growth in children is attributed to increasing levels of IGF-1. Administration of GH increases the maximum tubular phosphate reabsorption rate per GFR (TmP/GFR) in humans and canines, resulting in concomitant increases in serum phosphate levels [29, 49, 50]. Similar to the GH actions on GFR, these effects seem to be delayed (> 2h) and most likely mediated by IGF-1 (Fig. 1). *In vitro* perfusion studies, using isolated proximal convoluted tubules and *in vitro* studies using OK cells, showed that IGF-1 directly increases phosphate reabsorption via increase of protein levels of Na-Pi2a at the plasma membrane, which could be completely blocked by an anti-IGF-1R antibody [51–53].

#### Sodium and water

The sodium-retaining properties of GH, which are accompanied by an increased extracellular volume, were initially shown by Beck et al. using GH-containing pituitary extracts in healthy subjects and later confirmed in various studies using rhGH in humans and rodents [54–56]. In a randomized, 8-week trial GH significantly increased extracellular water mass in male and female athletes by 10.2% (1.8 kg) and 7.9% (1.2 kg) compared to controls, which was significantly associated with more frequent reports of swelling [54]. Initial studies suggest that the GH-induced sodium and water retention is mediated by the renin-angiotensin-aldosterone system (RAAS), which could not be confirmed [57, 58]. IGF-1 acts as an antinatriuretic in healthy subjects and in children with GHR-defects, suggesting that its sodium-retaining properties may be at least partly mediated by IGF-1 [59, 60]. The sodium-retention properties of IGF-1 can be blocked by amiloride, suggesting the involvement of the epithelial sodium channel (ENaC) [61]. Indeed, recent elegant studies in humans and collecting duct cell lines showed that IGF-1 regulates sodium reabsorption in the distal nephron by activation of the IGF-1R, which results in upregulation of the ENaC [25, 26]. These effects were shown to be independent of aldosterone, a known regulator of ENaC-dependent sodium reabsorption [25, 26]. This concept is also supported by studies in rats with GH-producing tumors showing increased natriuretic response when treated with amiloride, which is a selective ENaC blocker, whereas natriuretic response was decreased by furosemide, which acts on the loop of Henle (Fig. 1) [11]. Also, an increased natriuretic response to amiloride, but not to furosemide, was recently demonstrated in acromegalic patients, which further supports the concept that GH increases ENaC activity [62].

Viengchareun et al. proposed that GH may also directly regulate ENaC by stimulating the transcription of the α-subunit of ENaC via the JAK2/STAT5 and MAPK pathways [63]. This was based on *in vitro* studies showing that GH can increase binding of phospho-STAT5 to the response element of the promotor of the *SCNN1A* gene encoding the αENaC subunit [63]. Thus, GH and IGF-1 may act synergistically to stimulate renal sodium reabsorption. However, direct proof that GH activates ENaC-dependent sodium transport in distal tubules is lacking. Taken together, studies in healthy subjects suggest that pharmacological doses of GH and IGF-1 promote sodium retention with consecutive edema formation, which is transient in most subjects.

#### Calcium and vitamin D metabolism

Calcium hemostasis is mainly regulated by activated vitamin D (calcitriol) and PTH. Both, GH and IGF-1 lead to a positive calcium balance via their effects on vitamin D metabolism, which is especially important in children due to the demands of their growing skeleton, whereas their effects on PTH are unclear. GH stimulates renal calcitriol synthesis in humans and rodents, which was shown to be mediated by IGF-1 via stimulation of 1α-hydroxylase in the proximal tubule (Fig. 1) [64–68]. Subsequently, calcitriol increases intestinal calcium absorption by stimulation of the epithelial calcium channel TRPV6 [69, 70] and stimulates renal calcium absorption in the distal renal tubule by upregulating the expression of the epithelial calcium channel TRPV5 [71]. The latter is supported by studies in acromegalic patients showing enhanced renal calcium absorption in the distal tubule, but not in Henle’s loop [72]. Both mechanisms promote a positive calcium balance [73].

#### Acid–base hemostasis

Metabolic acidosis is rarely reported in children with GH-deficiency [74]. However, retrospective biochemical analysis in short children with GH-deficiency and children with other causes of growth failure, revealed significant lower mean serum bicarbonate concentrations in patients with GH-deficiency [74]. In addition, a significant increase was noted in GH-deficient children during treatment with rhGH, suggesting that GH and/or IGF-1 affect acid-base hemostasis. This is further supported by studies in hypophysectomized rats demonstrating metabolic acidosis, which can be corrected by GH treatment [75]. GH stimulates the production of ammonia in canine renal proximal tubule segments, suggesting that it may be a regulator of net acid secretion [76]. In addition, studies in humans indicate that GH also stimulates distal tubular Na^+^ absorption which may be essential for the GH-induced distal nephron proton secretion (Fig. 1) [77]. Treatment with GH in humans with NH_4_Cl-induced chronic metabolic acidosis restored plasma bicarbonate levels, which was associated with an increase in renal net acid secretion. However, these effects were completely blunted in NaCl-restricted subjects, despite GH-stimulated renal ammonia production as suggested by increases in urine pH, and could be restored after changing back to a normal NaCL diet. Thus, the effects of GH on net acid secretion are dependent on the availability of a surfeit of a Na^+^ tubular reabsorption. Taken together, the current available data suggest that GH and/or IGF-1 regulate acid-base hemostasis by stimulation of ammonia production in the proximal renal tubule and/or by a Na-dependent mechanism in the collecting duct, resulting in enhanced proton secretion.

#### Gluconeogenesis

Under euglycemic conditions, gluconeogenesis in proximal tubular cells is almost exclusively used by the kidney itself, mainly by medullary cells. However, during fasting conditions gluconeogenesis by the kidneys amounts to up to 50% of endogenous glucose production in order to maintain euglycemia [78]. During the fasting state, GH-secretion from the pituitary glands is markedly stimulated, whereas insulin and IGF-1 secretion is reduced. GH was shown to enhance renal gluconeogenesis in proximal tubular cells via activation of the GHR, independent of IGF-1 [10, 79]. Thus, in the fasting state GH-induced renal gluconeogenesis is essential in order to maintain euglycemia (Fig. 1).

#### Klotho

The antiaging protein α-Klotho is a 130 kDa membrane-bound protein mainly synthesized in the kidneys and acting as a coreceptor for FGF23, thereby mediating its renal effects with respect to phosphate handling and calcitriol synthesis [80]. FGF23 acts in opposition to GH by increasing phosphaturia via suppression of Na-Pi 2a and 2c expression in proximal tubules and decreasing renal calcitriol synthesis, whereas it shares its calcium-sparing effects by enhancing renal calcium reabsorption in the distal tubule [81]. Klotho is mainly expressed in the distal renal tubule and, to a lesser extent, in proximal tubules. It also exists in a soluble form (sKlotho) which is generated by cleavage of the extracellular domain of α-Klotho, which circulates in the blood and also exerts systemic effects, including cardioprotective properties, which are still poorly understood [80, 82]. In general, the amount of circulating sKlotho is correlated to the expression of membrane-bound α-Klotho in the kidneys and, thereby, serves as an indicator of renal α-Klotho expression [80].

Treatment with rhGH has been shown to increase the circulating levels of sKlotho in healthy subjects and in adults with stage 3 CKD, as well as in patients with GH-deficiency [83, 84]. Similarly, rhGH-treatment was associated with higher sKlotho levels in children with CKD, irrespective of the underlying kidney disease [85]. Upregulation of sKlotho may possibly exert positive cardiac and vascular effects through enhanced FGF23-mediated NO release from small vessels, resulting in improved endothelial function (*vide supra*) [86]. These GH actions may also be partly mediated by IGF-1, as suggested by the findings of positive associations with sKlotho levels in GH-sufficient and GH-deficient pediatric patients with short stature [87, 88].

## Pathophysiology of GH

### GH hypersecretion

The effects of GH hypersecretion on the kidneys have been extensively evaluated in acromegalic patients and animals chronically exposed to excessive GH and IGF-1 levels, and in transgenic mice with GH or IGF-1 overexpression (Fig. 1) [89].

#### Acromegaly

##### Kidney morphology

Acromegaly is associated with renal hypertrophy in humans and rodents [1]. In a case control study, kidney length assessed by kidney ultrasound was significantly increased by approx. 5 cm (55%) and 2 cm (20%) in active and controlled acromegalic patients, respectively [90]. Kidney size rapidly normalizes within 3 to 6 months in acromegalic patients undergoing transsphenoidal surgery [91]. Systematic studies on renal histology in acromegalic patients are lacking. Rare cases, where acromegalic patients underwent kidney biopsy due to nephrotic syndrome or persistent proteinuria, revealed focal segmental glomerulosclerosis [92, 93]. In one acromegalic patient presenting with nephrotic range proteinuria and focal segmental glomerulosclerosis on kidney biopsy, proteinuria quickly normalized after tumor removal but returned 4 months later, but responded to prednisolone treatment [94]. Only moderate or nonglomerular hypertrophy was noted in acromegalic patients undergoing kidney biopsy. By contrast, rats bearing GH-secreting tumors displayed significant glomerular hypertrophy and interstitial edema but no tubular hypertrophy, resulting in a 1.6-fold increase in kidney weight in relation to body weight compared to controls [95]. However, GH levels are lower in acromegalic patients compared to animals that are not treated. Therefore, the discrepancy of the histological results obtained in acromegalic animals and humans may be, at least partly, due to significantly more pronounced GH excess in animals compared to humans.

##### Glomerular function

Acromegalic patients show glomerular hyperfiltration characterized by an approx. 15% increase in GFR and RPF compared to healthy subjects, which is reversible in most but not all patients by surgical removal of pituitary adenomas [62, 72, 96]. Persistent glomerular hyperfiltration is thought to contribute to the development of albuminuria in acromegalic patients undergoing delayed surgery [97, 98]. In the Baldelli study, microalbuminuria was reported in 55% of acromegalic patients and associated with hypertension, impaired glucose tolerance and diabetes [99].

##### Tubular function

Acromegalic patients show an increase in total body water and sodium and may present with overt edema. These changes are related to the sodium-retaining properties of GH and IGF-1 via ENaC in the renal distal tubules (*vide supra*) and can be reversed if patients undergo effective treatment of the GH-producing tumor [100]. Total (56% versus 50% of body weight) and extracellular body water (20% versus 15% of body weight), as well as exchangeable sodium, were shown to be increased in acromegalic patients compared to healthy subjects, whereas no differences were noted in intracellular water content [56, 101]. Plasma volume was also found to be increased in these patients [102–104]. The clinical consequences of these alterations are arterial hypertension, left ventricular hypertrophy and congestive heart failure, all contributing to the overall increased mortality in untreated patients [100]. Important to note, arterial hypertension is associated with an inferior outcome in these patients [100, 105]. In addition, diabetic acromegalic patients present with more pronounced left ventricular hypertrophy compared to nondiabetic patients [106].

Acromegalic patients often present with mild hyperphosphatemia despite increased GFR, due to increased TmP/GFR, which can be used as a comprehensive measure of disease status and can be reversed with treatment [107]. The underlying mechanisms include, IGF-1-induced up-regulation of the Na-Pi 2a cotransporter in the renal proximal tubules, as well as enhanced intestinal phosphate absorption, due to GH-induced increased calcitriol synthesis (*vide supra*). Patients often show serum concentrations toward the upper normal range in association with hypercalciuria [108]. These findings are most likely related to GH-induced calcitriol synthesis (*vide supra*), with consecutively increased intestinal calcium absorption, as calcitriol levels tend to be elevated in these patients [108]. In addition, enhanced calcium absorption in the kidneys was demonstrated in acromegalic patients which is most likely related to calcitriol-induced stimulation of TRPV5 expression in the distal renal tubules (*vide supra*) [109]. The altered calcium metabolism was thought to contribute to the increased skeletal fragility noted in acromegalic patients [110].

Circulating levels of sKlotho are markedly increased in acromegalic patients and return to normal in parallel with GH and IGF-1 levels after tumor removal [111, 112]. Therefore, using sKlotho levels as a measure of disease status in these patients was suggested [112]. Since α-Klotho is not expressed in pituitary adenoma, elevated sKlotho levels are most likely due to increased renal α-Klotho and consecutively, enhanced cleavage of its extracellular domain as the main source of sKlotho (*vide supra*) [112, 113].

#### GH/IGF-1 transgenic mice

Transgenic mice overexpressing human, bovine or rat *GH* genes exhibit excessive GH and IGF-1 concentrations resulting in a giant phenotype and organomegaly, including increased kidney weight even when related to increased body weight (Fig. 1) [114–119]. In addition, these animals develop glomerular hypertrophy, mesangial proliferation and matrix deposition at around 4 weeks. This is accompanied by podocyte injury, characterized by podocyte hypertrophy, foot process effacement and detachment of podocytes from the glomerular basement membrane. The consequences are albuminuria and progressive kidney failure, resulting in complete glomerulosclerosis and kidney failure at around 37 weeks of age. By contrast, transgenic mice overexpressing IGF-1 show less pronounced glomerular hypertrophy and do not develop glomerulosclerosis [120, 121]. In order to dissociate the contributions of GH and IGF-1-excess from the development of kidney damage, Blutke et al. compared the renal phenotype of IGF-1-deficient mice (*I* ^-/-^), IGF-1-deficient GH-transgenic mice (*I* ^-/-^ /*G*), GH-transgenic mice (*G*), and wild-type mice [122, 123]. Both, *G* mice and *I* ^-/-^ /*G* mice developed the aforementioned pathological glomerular changes. However, this was much less pronounced in *I* ^-/-^ /*G* compared to *G* mice. In addition, tubular hypertrophy was observed in *G* mice but not in *I* ^-/-^ /*G* mice. The authors concluded that GH-excess is able to promote severe glomerular damage in mice in the absence of IGF-1, whereas tubular hypertrophy observed in GH-transgenic mice is most likely mediated by concomitant IGF-1 excess. It must be mentioned that GH serum levels in GH-transgenic mice are orders of magnitude above those noted during rhGH-treatment in patients with GH-deficiency or CKD. Even in acromegaly, the GH-secretion rates are higher than the amount of rhGH given for treatment of growth failure [124].

### GH deficiency

In principle, the renal changes observed in patients with GH-deficiency mirror those observed in patients with GH-oversecretion. The availability of rhGH for treatment of GH-deficient patients enabled detailed analysis of the renal effects of GH in these patients.

#### Kidney morphology

GHR ^-/-^ mice present with reduced kidney size even after correction of reduced body weight. Also, kidneys in adult IGF-1 ^-/-^ mice are less than half the size compared to wild-type animals, and, IGF-1R ^-/-^ mice displayed severe intrauterine growth retardation and died immediately after birth, presenting hypoplastic organs including the kidneys [27, 125, 126].

Analogous to the experimental findings in rodents stated above, patients with GH-displaying mutations in the *GH*, *GHR*, and *IGF1* genes present with reduced kidney sizes [127–129]. Hypophysectomy in humans results in a reduction in kidney size by 20% after 5 months [130]. Treatment with rhGH in adult patients with childhood-onset GH-deficiency results in normalization of kidney size [131]. Equally, treatment with recombinant human IGF-1 (rhIGF-1) in children with severe IGF-1 deficiency, due to GHR-insensitivity, was shown to increase kidney length [132]. Unfortunately, no information is available on renal histopathological changes in GH or IGF-1-deficient patients.

#### Glomerular function

Humans with GH and IGF-1 deficiency display reduced GFR and RPF [133–135]. Similarly, hypophysectomy in humans results in a rapid decrease in GFR, even when patients receive hormone replacement for all pituitary deficiencies, except GH [130]. GH-replacement therapy restores GFR and RPF in some but not all studies, which is probably related to differences in duration of therapy and GH dose [134–136]. Treatment with rhIGF-1 in patients with GH-insensitivity also restores GFR [133].

#### Tubular function

GH-deficient children and adults display reduced sodium and total body water content and reduced extracellular and plasma volume compared to healthy subjects, while body fat mass is increased [137, 138]. All these changes can be normalized by rhGH-replacement therapy. Higher rhGH-doses may even result in acute fluid retention, leading to symptoms such as edema, weight gain, and carpal tunnel syndrome which usually disappears after dose-reduction or spontaneously [139]. Treatment with rhIGF-1 was shown to improve hydration status in children with GHR-insensitivity, further supporting the concept that the sodium and water-retaining properties of GH are at least partly mediated by IGF-1 (*vide supra*) [60].

Several randomized placebo-controlled trials on rhGH-replacement therapy in children and adults showed significant increases in serum calcium and urinary calcium concentrations in rhGH-treated patients. However, these effects were often transient, usually lasting 3–6 months, and no significant differences were noted at 9–12 months compared to controls [140–143]. Treatment with rhIGF-1 in children and adults with GHR-insensitivity resulted in increases in urinary calcium excretion, whereas serum calcium levels remained constant [60, 144]. These effects are most likely due to IGF-1-induced increased calcitriol synthesis, resulting in enhanced intestinal and renal calcium absorption (*vide supra*). Indeed, GH-replacement therapy, as well as treatment with rhIGF-1, increased serum calcitriol levels in GH-deficient patients [145, 146]. The latter was already observed within 3 days of treatment. Although theoretically this would have been expected (*vide supra*), no significant changes in intestinal calcium absorption were noted in GH-deficient patients receiving treatment with rhGH or rhIGF-1 [147].

As expected, GH-replacement therapy in children and adults improves TmP/GFR with consecutive increases in serum phosphate levels. In contrast to its effects on serum calcium, the antiphosphaturic effects of rhGH persist for at least 2 years [143, 148, 149]. By contrast, the antiphosphaturic effects of rhGH-treatment in children with inherited renal hypophosphatemia, i.e., X-linked hypophosphatemic rickets, were only transient, indicating the importance of other phosphaturic hormones, i.e., fibroblast growth factor 23, in this particular disease [50].

### GH in kidney disease

#### Unilateral and subtotal nephrectomy

Patients born with a solitary functioning kidney or undergoing unilateral nephrectomy show compensatory growth of the remaining kidney, with increased glomeruli and tubular sizes and increased single nephron GFR, resulting in normalization of total GFR. These adaptive mechanisms result in glomerular hyperfiltration and increased glomerular capillary pressure and are associated with kidney injury and hypertension in the long run [150]. There is evidence that alterations in the GH/IGF-1 axis contribute to this scenario, at least in the early stages. In adult rodents, unilateral nephrectomy results in an early but transient rise in the expression of IGF-1 mRNA in the remaining kidney and a transient rise in systemic GH-concentrations, whereas IGF-1R levels in the kidney are unaffected [151–153]. Therefore, locally synthesized, as well as increased circulating IGF-1, may contribute to the rapid compensatory kidney growth in mature rats after unilateral nephrectomy. By contrast, nephrectomy in immature rats was not associated with changes in circulating GH and IGF-1 levels but increased renal IGF-1 synthesis, which was limited to the first 7 days only, although compensatory growth continues for several months in this model [152, 153]. Therefore, there is no evidence that the GH/IGF-1 system is involved in compensatory kidney growth after this early period and other mechanisms including increased NO production and renal sympathetic nerve activity and/or activation of the RAAS may be the major factors maintaining compensatory kidney growth [150].

#### Chronic kidney disease

Chronic kidney disease results in profound alterations of the somatotropic hormone axis [154]. Fasting GH-concentrations were reported to be normal or even increased in CKD patients, caused by a prolonged plasma GH half-life, due to reduced GH clearance by the kidneys [42, 155]. By contrast, endogenous GH secretion was shown to be normal or even slightly decreased in pubertal children with CKD. CKD results in impaired GHR and GH signaling characterized by an altered GH-induced hepatic, as well as growth plate cartilage IGF-1 synthesis, due to a reduced expression of the GHR and/or a postreceptor signaling defect [156–158]. The JAK2/STAT5 signaling pathway is activated after binding GH to its receptor, followed by transcriptional activation of IGF-1 synthesis and induction of SOCS proteins acting as a negative feedback loop (*vide supra*). CKD results in an altered equilibrium between activation of IGF-1 and SOCS, toward a markedly increased SOCS synthesis, which is most likely due to CKD-associated chronic inflammation resulting in a state of GH-insensitivity [156, 157]. In addition, there is also evidence for IGF-1-insensitivity in CKD [159]. IGF-1 and IGF-2 serum concentrations were found to be within the normal range in children with CKD stages 1–4, while IGF-1 concentrations were slightly reduced, and those of IGF-2 slightly increased in patients with more advanced CKD [160]. Children with advanced CKD showed reduced somatomedin bioavailability, partly due to accumulation of IGFBPs (IGFBP-1, -2, -4, and -6), which are normally cleared by the kidneys. In addition, increased hepatic synthesis of IGFBP-1 and -2 was demonstrated in experimental uremia [161]. Finally, impaired cellular IGF signaling was demonstrated in experimental uremia [162].

In summary, the findings of impaired IGF-1 synthesis, together with modest elevation of GH-levels due to decreased renal clearance, and increased IGF plasma binding capacity, provides evidence of a multilevel, homeostatic failure in the GH-IGF-1 system in CKD, which has paved the way for studies proving the stimulatory growth effects of GH in short children with CKD [163]. A comprehensive review of the growth-promoting effects of rhGH and recommendations for its use in children with CKD was recently provided by Drube et al. [154]. In the following section, the effects of GH on kidney function in experimental uremia and in patients with CKD, before and after kidney transplantation (KTx), will be outlined.

##### Experimental studies

GH-induced hyperfiltration may have adverse effects on kidney function in CKD. Miller et al. investigated the effects of recombinant bovine GH (bGH) and rhIGF-1 on kidney function in normal and 5/6 nephrectomized rats by inulin and p-aminohippurate clearances over 10–17 days. GFR in 5/6 nephrectomized rats decreased to 17% one day after 5/6 nephrectomy, compared to controls, and slightly increased during the next 3 days [164]. However, in contrast to healthy rats showing sustained increases in GFR and RPF during treatment with bGH and rhIGF-1, no significant effects of these measures on GFR, RPF or filtration fraction were noted in 5/6 nephrectomized rats. Equally, a 7-day treatment with exogenous rat or rhGH, or rhIGF-1, failed to increase GFR in 5/6 nephrectomized rats in subsequent studies [165]. Since uremic animals also failed to show increases in GFR and RPF after amino acid infusion, known to enhance kidney function in healthy animals, the authors concluded that the vasodilatory eicosanoids are already maximally stimulated under baseline conditions in uremic animals and, consequently, renal reserve capacity is diminished in these animals.

Allen et al. demonstrated that prolonged treatment with rhGH for periods of up to 25 weeks did not adversely affect GFR compared to control animals [166]. Interestingly, kidney-to body-weight-ratio was normalized in rhGH-treated animals, due to much more pronounced glomerular hypertrophy in rhGH-treated rats when compared to uremic controls, as demonstrated by renal histological examination. In addition, both uremic controls and rhGH-treated uremic animals showed a significantly increased mean sclerotic index, compared to healthy rats, which was even more pronounced in rhGH-treated animals, suggesting that the beneficial effects of prolonged rhGH-treatment on kidney growth results in progressive glomerulosclerosis in rodents. Similar results were reported by Kawaguchi et al., investigating the renal effects of an 8-week treatment of various rhGH-doses (0.4, 2.0, and 10 IU/day) in subtotally nephrectomized rats [167]. Whereas low rhGH-doses did not result in significant changes in GFR or glomerular sclerosis index compared to controls, medium and high-dose rhGH-treatment resulted in a significantly lower creatinine clearance and glomerularsclerosis index. The translation of these results to the setting of chronic rhGH-treatment in children with CKD is hampered by the fact that the rhGH-dosages in these studies (approx. 3 mg/kg body weight (BW) per day) were 60-fold higher compared to those used for treatment of CKD-associated growth failure (0.05 mg/kg BW per day). Nevertheless, this data indicates that special attention should be paid to the use of rhGH in children with CKD, especially when given over prolonged periods [154].

#### Clinical studies

##### rhGH treatment

Three-day administration of rhGH at dosages comparable to those used for uremic growth failure did not increase GFR in adult CKD patients with a median baseline GFR of 21 ml/min/1.73 m^2^_,_ despite significant anabolic effects with respect to serum cholesterol levels and urea excretion rates [33]., Tönshoff et al. also showed no significant increases in GFR in uncontrolled studies on rhGH-treatment in children with CKD stages 3–4 (median GFR, 19 ml/min/1.73 m^2^) and after KTx (median GFR, 47 ml/min/1.73 m^2^) after a 6-week treatment period [168]. Maxwell et al. investigated GFR and RPF in 18 children with CKD (median GFR, 19 ml/min/1.73 m^2^) during their first year of rhGH-treatment [169]. GFR at 3h after first rhGH-injection, at day 8 and after 1 year remained unchanged, compared to baseline values. By contrast, a significant transient increase in RPF was noted on day 8 (96 (33–276) ml/min/1.73 m^2^ versus 77 (34–271) ml/min/1.73 m^2^). This data suggests that GH does not augment kidney function in humans with CKD stages 3–4 and may be due to GH-insensitivity associated with CKD and/or generally limited renal reserve capacity in these patients (*vide supra*). However, there is limited data suggesting that GH may increase GFR in patients with mild CKD. GFR was significantly increased after 1 week and 6 months of rhGH-therapy in a cohort of 16 pediatric KTx patients with CKD stages 2–3, but returned to baseline values thereafter, whereas no changes in RPF were noted (GFR: baseline 52 ml/min/1.73 m^2^, 1 week 57 ml/min/1.73 m^2^, 6 months 63 ml/min/1.73 m^2^) [170].

**Fig. 2:**
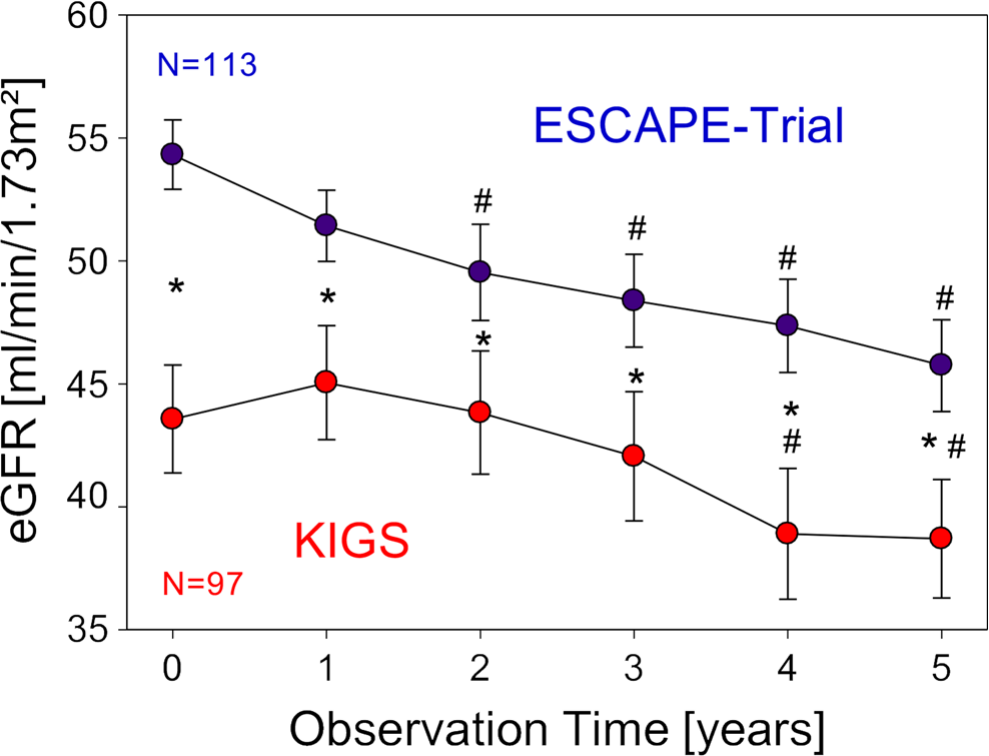
Changes in estimated glomerular filtration rate (eGFR) during the 5-year longitudinal follow-up. eGFR decline within 5 years was not significantly different between recombinant human growth hormone (rhGH)-treated (KIGS register) and non-rhGH-treated (ESCAPE trial) patients. Data is given as the mean± standard error of the mean (SEM). *Significant differences between groups, ^#^significant difference from baseline. Figure reproduced with permission from Mehls et al. [173].

Nine randomized controlled trials (RCTs) revealed no evidence of adverse effects of rhGH on estimated GFR (eGFR) over treatment periods of up to 2 years in children with CKD stages 2–4 [171]. Patients suffering from nephropathic cystinosis are especially prone to progressive CKD, despite treatment with cysteine-lowering agents. Wühl et al. investigated the evolution of eGFR values in children with nephropathic cystinosis started on rhGH-treatment and enrolled in a European multicenter trial, compared to that of a historical control group with comparable ages and eGFR [172]. The decline in mean eGFR was comparable in rhGH-treated patients compared to controls, irrespective of concomitant cysteamine treatment. Mehls et al. assessed the effects of rhGH on eGFR in children with CKD stages 2–4, using data from two large prospective clinical studies involving children with (KIGS registry) and without rhGH-therapy (ESCAPE trial) [173]. In both studies, eGFR was assessed cross-sectionally in yearly intervals for up to 10 years and longitudinally for 5 years. Overall, patients showed only a mild progression in CKD during the observation period. The mean decline in eGFR at 5 years compared to baseline did not significantly differ between groups (KIGS, −5.8 ml/min/1.73 m^2^; ESCAPE, −8.6 ml/min/1.73 m^2^, *p* = 0.17) (Fig. 2). In both patient cohorts, the change in eGFR after 5 years was significantly associated with age and height at baseline. It must be mentioned that rhGH-treatment is known to improve muscle mass in CKD which may have resulted in an increase in serum creatinine and, consequently, a decrease in eGFR in the KIGS cohort. However, this further supports the findings that rhGH-therapy does not lead to accelerated progression of CKD in children. Recent experimental studies suggest that the safety of prolonged rhGH-treatment in CKD may be, at least partly, due to GH-insensitivity in the kidney in these patients. Landau et al. noted reduced expression of the GHR in the kidneys of 5/6 nephrectomized animals, which was associated with elevated pro-inflammatory cytokines, suggesting that GH-insensitivity in uremia is a general phenomenon and not restricted to the liver and growth plates (*vide supra*) [174].

Concern has been raised that rhGH may stimulate the immune system in KTx patients and thereby promote acute rejection episodes with the consequence of poor graft outcome in children. However, a recent meta-analysis of 5 RCTs on rhGH-treatment after KTx including 401 children revealed no increased risk for acute rejection episodes (risk ratio 1.56; 95% CI 0.97–2.53, *p* = 0.07) or increased decline in eGFR (delta eGFR at 1 year, 3.27 ml/min/1.73 m^2^; 95% CI −3.54–10.09, *p* = 0.35) [175].

Taken together, there is no evidence that rhGH-treatment accelerates the decline of kidney function in children with CKD or promotes acute rejection episodes after KTx. when using the recommended rhGH standard dose (0.045–0.05 mg/kg per day, s.c.). Nevertheless, a European guideline recommends that eGFR should be monitored in at least 3-month intervals in children with CKD started on rhGH treatment, and that treatment should be discontinued in patients presenting with an unexplained decline in eGFR [154].

##### rhIGF-1 treatment

O’Shea et al. investigated the renal effects of a 3-day rhIGF-1 treatment (100 μg/kg twice daily) in 4 adult patients with advanced CKD (GFR 21–55 ml/min/1.73 m^2^) showing significant increases of both GFR and RPF in all patients, which was associated with a small increase in kidney size [176]. The same group later investigated whether a longer rhIGF-1 treatment (20 days) in patients with more advanced CKD (GFR ≤ 21 ml/min/1.73 m^2^) would have similar results [177]. Although, there was an initial rise in GFR (32%), RPF (28%) and the percentage of tubular reabsorption of filtered phosphate at day 4, all these parameters returned to baseline levels after 13 or 20 days of rhIGF-1 treatment, but the patients developed severe side effects, including jaw pain, nasal congestion and Bell’s palsy, requiring discontinuation of rhIGF-1 treatment in 4 of 9 patients. In a subsequent RCT of the same group, a lower IGF-1 dosage (50 μg/kg twice daily) and intermittent treatment protocol, with cycles of 4-day treatment interrupted by 3 days off treatment, in patients with stage 5 CKD (GFR 6–15 ml/min/1.73 m^2^) was used [178]. RhIGF-1 resulted in significant, sustained increases in GFR (42–81%) over a treatment period of 45 days. One patient showed mild edema and two patients displayed irritation at the injection site. Several patients in whom treatment was continued remained off dialysis for up to 18 months after initiation of rhIGF-1 treatment. Interestingly, GFR remained increased even 14 days after cessation of rhIGF-1 therapy compared to placebo-treated controls, suggesting that rhIGF-1 resulted in renal hypertrophy, since the hemodynamic effects should have worn off within 14 days of cessation. However, adequately powered RCTs are needed to prove the long-term efficacy and safety of rhIGF-1-treatment to enhance GFR in patients with advanced CKD.

#### Acute kidney injury

##### Experimental studies

Ischemic acute kidney injury (AKI) in rodents and humans results in preferential damage of the distal segment of the proximal tubule (S_3_-segment) and the thick ascending limb of the loop of Henle, indicated by loss of brush borders, cell necrosis and apoptosis, since this part of the nephron heavily depends on medullary blood flow in order to preserve oxygen supply [179]. Observations in rodents showed that the proximal tubule has IGF-1Rs and is responsive to IGF-1, and that renal IGF synthesis, including the collecting duct, is transiently increased in animal models of postischemic AKI, thereby providing the rationale for the use of rhGH or rhIGF-1 in AKI in order to improve kidney regeneration [3].

Miller et al. performed the first study showing evidence that treatment with rhIGF-1 improves renal recovery after postischemic injury in rodents [180]. Rats treated with IGF-1, either 24 h before ischemic AKI, immediately after or 24 h after, showed accelerated recovery of GFR and histopathological changes in damaged proximal tubular epithelia, as well as improved mortality. These results were later confirmed by several other groups using various animal models of ischemic or toxic AKI [181–183]. The beneficial effects of IGF-1 treatment in AKI may be due to the following: (1) its stimulatory effect on GFR via increased renal blood flow (*vide supra*), which prevents the obstruction of tubules by cellular debris and thereby limiting the extent of kidney injury; (2) its positive anabolic effects, including reduced protein breakdown; (3) its stimulatory effect on DNA synthesis in regenerating renal tubular cells; and (4) a reduced apoptosis rate after reperfusion [2].

Due to the positive effects of IGF-1 on kidney regeneration after AKI, Matejka and Bengtsson investigated the effects of high-dose rhGH-therapy in an animal model of postischemic AKI [184]. Rats underwent renal ischemia for 45 min. After reperfusion, the animals were started on rhGH-treatment (2 mg/day). Although rhGH-treatment partly restored reduced expression of GHR and IGF-1 in the kidneys and resulted in profound metabolic effects, including improved weight gain and lower urea serum levels, there was no difference with respect to renal recovery and mortality compared to placebo-treated controls. These negative results suggest severe GH-insensitivity in AKI, which cannot be overcome by exogenous treatment with rhGH, which contrasts with the results of rhGH-treatment in animals and humans with CKD.

##### Clinical studies

The effects of treatment with rhGH on renal recovery in patients with AKI was investigated in two RCTs. Franklin et al. showed that rhGH given after surgical interventions resulting in transient interruption of renal blood flow prevented the decrease in GFR noted in placebo-treated controls [185]. However, the incidence of AKI in this study was too low to draw meaningful conclusions on whether IGF-1 ameliorates the course of AKI. A second large placebo-controlled trial in patients with established AKI was terminated early, since an interim analysis showed no beneficial effects of rhIGF-1 treatment [3]. The negative results of the latter study may, at least partly, be due to the large heterogeneity of causes of AKI (trauma, hypertension, surgery, drugs or sepsis) and/or the delayed initiation of rhIGF-1 treatment, i.e., after established AKI.

#### Diabetic nephropathy

Patients with poorly controlled type 1 diabetes mellitus (T1DM) present with markedly enhanced pituitary GH-secretion rates, probably related to decreased hypothalamic somatostatin tone and exaggerated GH-responses to physiological and pharmacological stimuli [186, 187]. The primary underlying pathomechanism is most likely a decreased hepatic GHR expression related to insulin deficiency, resulting in GH-resistance and impaired hepatic IGF-1 synthesis, which stimulates pituitary GH-secretion by a feedback mechanism [188, 189]. In addition, hypoinsulinemia in T1DM stimulates hepatic synthesis of IGFBPs, resulting in increased IGF-binding capacity and, consequently, reduced IGF-1 bioactivity which further stimulates GH-secretion [190–192]. Since GHR expression in the kidney is unaltered in T1DM, elevated circulating GH-concentrations can impact on the kidney. Indeed, patients with T1DM present with increased GFR (25–50%), glomerular hypertrophy and increased kidney size during the early course of the disease, which may promote the development of diabetic nephropathy [193, 194]. The latter is characterized by glomerular hyperfiltration, glomerular/tubular hypertrophy, and thickening of the glomerular basement membrane and mesangial matrix expansion/proliferation, resulting in increased glomerular permeability with consecutive albuminuria and progressive CKD [195]. In addition, loss of podocytes, either due to apoptosis and/or podocyte detachment from the glomerular basement membrane, was shown to be an early event in the development of diabetic nephropathy in humans and various animal models of diabetic nephropathy [196–198].

The first evidence that excessive GH-levels are involved in the pathogenesis of diabetic microangiopathy came from observations in diabetic patients undergoing pituitary ablation, which resulted in improvement of diabetic retinopathy in some but not all patients [199]. Urinary GH and IGF-1 levels are associated with the presence of microalbuminuria in patients with T1DM [200, 201]. In addition, urinary IGF-1 concentrations are associated with kidney size and microalbuminuria in these patients [201].

As outlined above, GHR and IGF-1R are highly abundant in glomerular cells, including podocytes and mesangial cells, and can be activated by GH and IGF-1, respectively. Excessive levels of GH were shown to induce glomerular hypertrophy and glomerulosclerosis, which may be directly caused by GH or mediated by IGF-1 (*vide supra*). Alongside their humoral actions, both GH and IGF-1 can target the kidney in an autocrine and/or paracrine manner. Although GHR abundance in the liver in diabetic rats is reduced, GHR expression in kidneys was shown to be evenly increased, suggesting tissue-specific expression of GHR [202]. Reduced circulating IGF-1 and growth failure were also reported in T1DM patients, while IGF-1 concentrations in renal tissue were shown to be increased, suggesting increased local synthesis and/or IGF-1 sequestration from the circulation [203, 204]. In addition, increased IGFBP levels were reported in kidneys from diabetic rats, which may also contribute to increased renal IGF-1 levels [205]. Finally, GH signal transduction and IGF-1R expression were shown to be increased in streptozotocin-induced diabetic rats [206].

Further evidence comes from interventional studies in animal models of diabetic nephropathy targeting the GH/IGF system. Treatment with GH antagonists or somatostatin analogs, as well as genetic disruption or pharmacological blockade of the GHR, resulted in amelioration of experimental diabetic nephropathy, including normalization of diabetes-associated renal hypertrophy and glomerular enlargement, as well as albuminuria [203, 207–210]. These studies support the concept that a functional GHR is essential for the development of diabetic nephropathy in murine models of T1DM. Two studies in patients with T1DM further support this concept. Infusion with a somatostatin analog, SMS 201-995, acutely reduced GFR and RPF in uncomplicated patients with T1DM [211]. In addition, a significant reduction in kidney volume and hyperfiltration was noted after a three-month treatment in T1DM patients treated with octreotide, a somatostatin analog, compared to placebo-treated patients [212].

GH-induced increased podocyte permeability to proteins was originally thought to be related to increased expression of ZEB2, resulting in downregulation of E- and P-cadherins (*vide supra*). The same group recently revealed a new mechanism of GH-induced podocyte injury. They could demonstrate that GH increases expression of transforming growth factor-beta-induced protein (TGFBIp) in cultured podocytes with consecutively enhanced TGFBIp secretion in cell supernatant [195]. TGFBIp expression was also increased in renal tissue from patients with diabetic nephropathy. Both treatment with GH and TGFBIp increased podocyte migration, as well as podocyte permeability to albumin across podocyte monolayers. Finally, renal histological studies in animals treated with GH showed glomerular mesangial matrix expansion and increased apoptosis of podocytes. Taken together, these studies suggest that GH induces TGFBIp in podocytes, which may contribute to podocyte depletion in diabetes mellitus. The authors speculated that targeting TGFBIp may be an effective measure in preventing podocyte loss and, thereby, ameliorate progression of kidney disease in patients with T1DM. Nishad et al. revealed an additional potential mechanism of GH-induced podocyte injury [213]. By use of cultured immortalized podocytes and mouse models, they could demonstrate that GH excess activates Notch1 signaling in podocytes resulting in podocyte loss. Pharmacological inhibition of Notch1 prevented GH-induced glomerular fibrosis, glomerular basement membrane thickening and albuminuria *in vivo*. Upregulated Notch signaling was also noted in kidney biopsy sections from patients with diabetic nephropathy. These studies suggest that inhibition of GH-induced Notch1 signaling may be a promising measure in preventing diabetic nephropathy.

Important to note, GH was demonstrated to have nephroprotective effects as well [214]. GH treatment protected cisplatin-induced nephropathy in rats by reversing upregulated oxidative stress and inflammatory biomarkers (high mobility group box protein-1 and nuclear factor kappa B). Therefore, the results of the abovementioned studies should not be translated to other clinical settings.

Taken together, GH excess associated with poorly controlled T1DM induces podocyte injury, characterized by podocyte hypertrophy, apoptosis, dedifferentiation of podocytes (epithelial–mesenchymal transition) and/or cross-linking of the basement membrane resulting in increased podocyte permeability to albumin and detachment of podocytes from the glomerular basement membrane (Fig. 1). Therefore, GH excess may be an important promoter of diabetic nephropathy in poorly controlled T1DM patients.

#### Nephrotic syndrome

Clinical and experimental data have demonstrated profound disturbances of IGF-1/-2 and its binding proteins in the circulation and urine, which were originally thought to be due to increased glomerular permeability and urinary losses of these proteins [215, 216]. In pediatric patients with nephrotic syndrome and normal eGFR, mean standardized values of IGF-1 were found to be significantly decreased (−0.53 SD), whereas IGF-2 levels were increased (0.68 SD) [215]. Urinary excretion rates of IGF-1 and 2 were 5-fold increased, compared to healthy controls. Although, IGFBP-1 and 2 urinary excretion rates were 12-fold and 2-fold increased, the respective serum concentrations of these peptides were markedly increased (IGFBP-1, 2.05 SD; IGFBP-2, 5.97 SD). Fast liquid chromatography (FPLC) analysis of nephrotic serum revealed a decreased 150 kDA IGFBP ternary complex due to reduced intact IGFBP-3, whereas IGFBP-3 low molecular weight fragments were markedly increased, suggesting increased proteolytic degradation of IGFBP-3. In both patients and controls only IGBP-3 fragments but no intact IGFBP-3 could be detected, which was associated with the degree of proteinuria. By contrast, serum levels of the ALS and GHBP were found to be normal in nephrotic patients. Taken together, in children with nephrotic syndrome IGF-1 levels are decreased and high-affinity IGFBPs (mainly IGFBP-1/-2) are markedly increased, resulting in an excess of unsaturated IGFBPs which may inhibit IGF action on target tissues by enhanced IGF-binding and/or competition with the IGF-1R. The authors hypothesized that these alterations may contribute to catabolism and impaired growth in children suffering from long-standing nephrotic syndrome.

Studies in nephrotic rats performed by Hirschberg and coworkers revealed deep insights into the pathophysiology of the IGF/IGBP system in nephrotic syndrome. By use of micropuncture studies in rats with adriamycin-induced nephrotic syndrome, they could demonstrate that IGF-1 serum concentrations are markedly reduced and IGF-1 is highly ultrafiltrated together with IGFBP-2 appearing in proximal tubular fluid [217]. In line with the findings in humans, IGFBP-2 serum concentrations were markedly increased in nephrotic animals despite excessive urinary losses due to increased hepatic synthesis, whereas hepatic synthesis of IGFBP-3 was unchanged compared to controls. Western immune analysis suggested *in vivo* proteolysis of intact IGBF-3, resulting in reduced binding to IGFBP-3 and increased low-molecular weight IGFBP-3 fragments.

In their subsequent *in vitro* studies using cultured proximal tubular cells, they could demonstrate that proximal tubular fluid from nephrotic rats activates IGF-1R, suggesting that filtered IGF-1 is bioactive [218]. In addition, the tubular ultrafiltrate from nephrotic rats stimulated collagen types I and IV synthesis in cultured tubular cells, suggesting that the excessive losses of IGF-1 in the nephrotic state may promote tubular-interstitial fibrosis and thereby, progressive kidney disease in patients with persistent nephrotic syndrome [219].

### GH in patients with healthy kidneys

Concern has been raised that children treated for short stature not due to kidney diseases, receiving long-term rhGH-treatment may develop CKD in the long run. A recent, large, prospective study evaluated eGFR, blood pressure and microalbuminuria at 6 months, 2 years and 5 years after cessation of rhGH-treatment in adults born small for gestational age (SGA) [220]. A significant decrease in eGFR was noted at 6 months only. Mean blood pressure values and albuminuria did not differ in patients with prior rhGH-treatment compared to healthy controls born SGA during the follow-up period. This confirms previous clinical studies and registry analyses on the renal safety profile of rhGH-treatment in children with short stature of nonrenal origin [221–224].

## Summary and conclusion

The GH/IGF-1 system is an important regulator of kidney function, including glomerular hemodynamics, renal gluconeogenesis, and tubular handling of phosphate, sodium, water, and calcium. The latter is mediated by GH/IGF-1-induced renal synthesis of 1,25 (OH)_2_ vitamin D_3_. Treatment with rhGH increases GFR and RPF in healthy subjects via IGF-1-induced synthesis of the endogenous vasodilator NO. GH-excess in acromegalic patients and GH-transgenic animals can result in glomerular hyperfiltration, renal hypertrophy, and glomerulosclerosis. Likewise, elevated GH in patients with poorly controlled T1DM was also shown to promote diabetic nephropathy in these patients. Therefore, concern was raised that long-term treatment with rhGH in short children with CKD may accelerate CKD progression. However, clinical and experimental studies demonstrate the long-term safety of this treatment in subjects with impaired and normal kidney function. This discrepancy is probably due to the CKD-associated GH insensitivity of the kidneys, preventing GH actions on the glomerular cells and/or the considerably lower GH exposure in rhGH-treated patients, compared to GH excess in acromegalic patients and GH transgenic animals. Finally, GH was shown to increase the renal synthesis of the antiaging hormone Klotho in healthy subjects and patients with CKD, indicating that GH treatment may have beneficial effects on the cardiovascular system. However, this has to be proven in adequately designed clinical studies.
